# Reactivation of latent tuberculosis infection induced by cabazitaxel in a patient with prostate cancer: A case report

**DOI:** 10.1097/MD.0000000000018436

**Published:** 2019-12-20

**Authors:** Mamoru Hashimoto, Takafumi Minami, Mamoru Hamaguchi, Saizo Fujimoto, Tomoki Takahashi, Takashi Kikuchi, Shogo Adomi, Eri Banno, Takayuki Ohzeki, Nobutaka Shimizu, Yasunori Mori, Masahiro Nozawa, Kazuhiro Nose, Kazuhiro Yoshimura, Hirotsugu Uemura

**Affiliations:** Department of Urology, Kindai University Faculty of Medicine, Osaka-Sayama, Osaka, Japan.

**Keywords:** cabazitaxel, calcified nodule, latent tuberculosis infection, prostate cancer, reactivation

## Abstract

**Rationale:**

Latent tuberculosis infection (LTBI) describes the dormant state of tuberculosis (TB), in which persistent immune-related interaction between TB and T-cells maintain its state. Cabazitaxel (CBZ) is reported to improve overall survival in patients with castration-resistant prostate cancer (CRPC) after progression observed in regimens including docetaxel. CBZ is known for severe myelosuppression; however there is no recommendation for the treatment of LTBI before CBZ treatment. To the authors’ knowledge, this is the first report to describe reactivation of LTBI induced by CBZ.

**Patient concerns:**

A 75-year-old Japanese male with a medical history of TB since 16 years of age had been treated for prostate cancer (PC) (initial prostate-specific antigen 532 ng/ml; cT4N1M1b; Gleason score4+4) with androgen deprivation therapy, abiraterone, and docetaxel. Calcified nodules and radiological findings of LTBI were present in the upper right lobe since the diagnosis of PC. After progression was observed during these treatments, CBZ was administered combined with pegfilgrastim, long-acting granulocyte colony-stimulating factor (G-CSF). Seven days after the third course of CBZ, he was admitted to the authors’ hospital to treat febrile neutropenia (FN). High fever persisted even after myelosuppression had recovered. Computed tomography (CT) revealed distribution of small nodules in the bilateral lungs, for which miliary TB was included in the differential diagnosis. T-Spot, interferon-gamma-release assay, and bronchoscopy yielded no significant findings; however, sputum and urine culture confirmed the diagnosis of TB.

**Diagnosis:**

CT, sputum and urine culture confirmed the diagnosis of miliary TB.

**Interventions:**

The patient was treated with anti-bacterial therapy (cefepime) on hospital admission, which was not effective. After the diagnosis of miliary TB was confirmed, anti-TB drugs, including isoniazid, rifampicin, pyrazinamide and ethambutol, were administered.

**Outcomes:**

Despite anti-TB therapy, high fever persisted and radiological findings worsened. Fifty days after the third course of CBZ, the patient died of respiratory dysfunction caused by progression of miliary TB.

**Lessons:**

Management of LTBI is needed in cases of radiographic findings of LTBI and medical history of TB before CBZ treatment, despite the rarity of LTBI reactivation in patients with PC.

## 1 Introduction

The term LTBI has been used since the American Thoracic Society and Centers for Disease Control and Prevention issued recommendations for targeted screening and treatment of LTBI intended to eradicate TB. They supported the concept of concentrating resources for screening and treatment of LTBI in individuals with significant risk factors for reactivation of LTBI. Risk factors included several cancers (hematological malignancies, lung cancer, and head and neck cancers), immune suppressive states (HIV-positivity, uncontrolled diabetes mellitus, organ transplant, substantial and long-term administration of steroids), and nodules and fibrotic scars on chest radiography, which are signs of former TB infection; however PC was not included.^[1]^ CBZ has been reported to improve overall survival in patients with CRPC after progression observed in regimens including docetaxel.^[2]^ CBZ is known for adverse events such as severe neutropenia, and grade 3–4 lymphopenia has been reported in up to 69% of patients.^[3]^ LTBI is the dormant state of TB, with T-cells playing a key role in maintaining dormancy. T-cells activated in the presence of TB mediate several types of immune cells, which then form granulomas and confine TB. These immune cells constantly infiltrate the granuloma, if the balance is disturbed by certain immune suppression, disintegration of the granuloma results. Radiographic findings of LTBI are reflected by the formation of granuloma; therefore these findings may be included as risk factors.^[1,4,5]^ Thus, careful management of LTBI in patients with typical radiological findings of former TB infection is needed before initiation of CBZ therapy, despite PC not being a risk factor. To our knowledge, we report the first case of reactivation of LTBI induced by CBZ. The patient had a history of TB in his teens, and radiological findings of the former TB infection were evident since the diagnosis of his PC. Patient has provided written informed consent for publication of the case before the administration of CBZ.

## 2 Case report

**Figure 1:**
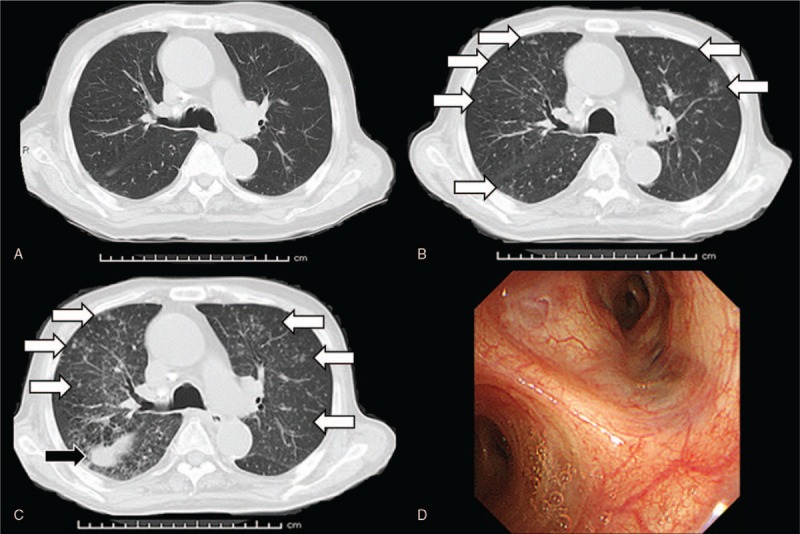
Compared with the findings of chest computed tomography before cabazitaxel treatment (A), small nodules distributed around the bilateral lungs (white arrows) emerged 15 days after the third course of cabazitaxel therapy (B). Findings of diffusely distributed small nodules worsened (white arrows) and pleural effusion (black arrow) was newly observed 23 days after the third course of cabazitaxel therapy (C). Bronchoscopy of the respiratory system did not yield any significant findings (D).

**Figure 2:**
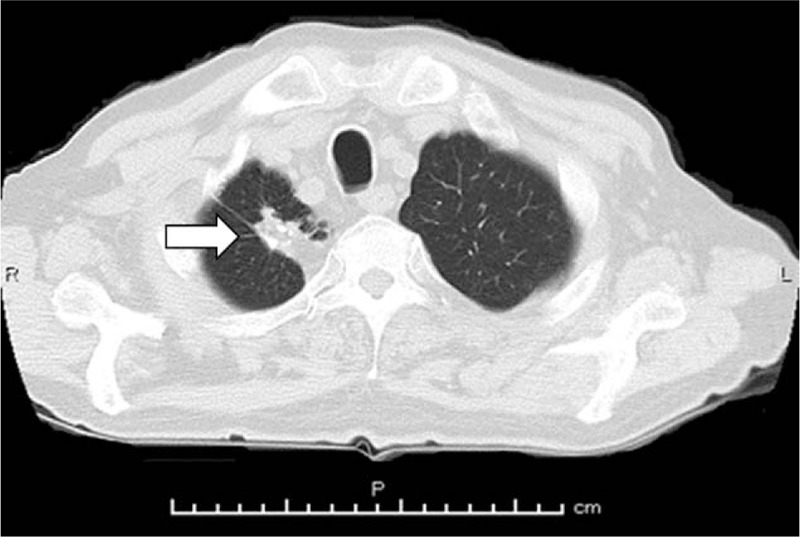
Computed tomography revealing calcified nodule (white arrow) in the right upper lobe of the lung, long before or, at least since, the diagnosis of prostate cancer.

A 75-year-old Japanese male (height, 157 cm; body weight, 50 kg; body surface area, 1.48 m^2^) with a medical history of TB since 16 years of age had been treated for PC (initial prostate-specific antigen 532 ng/ml; cT4N1M1b; Gleason score 4+4) with androgen deprivation therapy, abiraterone, and docetaxel. He smoked 20 cigarettes per day since he was 20 years of age. After a total of 7 courses of monthly docetaxel (75 mg/m^2^), CBZ was administered at full dose (25 mg/m^2^). Pegfilgrastim (3.6 mg), G-CSF medication with long-acting effectiveness, was co-administered the day after each CBZ treatment. The dosage of CBZ was reduced from full to three-quarters since the beginning of second course due to FN. Despite the reduction in dosage, he was admitted to hospital to treat his FN 7 days after the third course of CBZ. Three-weekly schedule of CBZ could not be maintained due to the adverse event. Blood tests revealed white blood cell count of 580/L, with grade 4 neutropenia and grade 3 lymphopenia (160/L and 340/L, respectively). Anti-bacterial therapy (cefepime) was started to treat his FN. High fever persisted for an extended period, even after myelosuppression recovered. Compared with the chest CT before CBZ treatment (Fig. 1A), small nodular shadows distributed around the bilateral lungs emerged 15 days after the third course of CBZ (Fig. 1B), at which time miliary TB was included in the differential diagnosis. A total of 3 sputum Ziehl-Neelsen (ZN) stains performed every 24 hours for the detection of mycobacterium were all negative. High fever (approximately 39°C) persisted; therefore CT was performed once again 23 days after the third course of CBZ. Results revealed progression of a small nodular shadow and pleural effusion emerged (Fig. 1C). Bronchoscopy with bronchoalveolar lavage (BAL) performed by a pulmonologist yielded no significant findings in the respiratory tract (Fig. 1D) and ZN stain of BAL fluid was negative. T-Spot, interferon-gamma-release assay, was also negative. However, polymerase chain reaction testing of BAL fluid was positive, and mycobacterium culture of sputum and urine submitted several days previously, confirmed the diagnosis of TB with sensitivity to anti-TB drugs. Anti-TB drugs, including isoniazid, rifampicin, pyrazinamide and ethambutol were started 30 days after the third course of CBZ to treat miliary TB. Fifty days after the third course of CBZ, the patient died of respiratory dysfunction caused by progression of miliary TB. CT findings of calcified nodules, a typical finding in LTBI, were observed since the diagnosis of PC (Fig. 2).

## 3 Discussion

To our knowledge, there have been no reports of reactivation of LTBI caused by CBZ; the same is similarly true for docetaxel, another approved chemotherapy for the treatment of PC. Cancers such as hematological malignancies, lung cancer, and head and neck cancers, have been categorized as risk factors for reactivation of LTBI; however, PC has not been included.^[1]^ Despite the lack of evidence, we encountered a case involving reactivation of LTBI induced by CBZ and the patient died of activated TB. Dose escalation of CBZ is reported to be linked to severe myelosuppression.^[6]^ Despite dose reduction of CBZ and combined use of pegfilgrastim, our patient developed FN and reactivation of LTBI. The reservoir of hematopoietic stem cells is reported to malfunction in elderly individuals.^[7]^ As such, management of LTBI is especially important for elderly individuals who undergo CBZ treatment. In terms of diagnosis of TB, CT findings of distributed nodules around the bilateral lungs, highly suspicious for miliary TB, were observed; however ZN-staining and T-Spot assay were negative. Although T-Spot is a useful method, which only requires a simple blood sample, the results are not totally reliable due to its sensitivity and specificity.^[8]^ In patients with miliary TB, urine culture is often reported to be positive,^[9]^ and was one of the definitive findings in our case. Therefore, in addition to sputum culture, urine culture should be performed as early as possible if miliary TB is suspected.

CBZ was developed to resolve the problem of resistance of tumor cells to the other taxanes, namely, docetaxel, and paclitaxel. One of the drug-resistance features of the taxanes is explained by overexpression of P-glycoprotein 1 (P-gp), which involves efflux of the drug through an ATP-binding cassette. The poor affinity of CBZ to P-gp results in its remaining inside cancer cells and exerts its antitumor effects stronger than the other taxanes.^[3,6]^ However, its toxicity especially severe myelosuppression, should be managed cautiously, similar to severe neutropenia managed with the use of G-CSF.^[10]^ Grade 3–4 lymphopenia has been reported in 69% of patients treated with CBZ.^[3]^ Moreover, pharmacodynamics of the prolonged half-life of CBZ (mean half-life, 77.3 ± 45.5 hours) expose patients to immunosuppressive states for extended periods.^[6]^ However, management options for lymphopenia, such as G-CSF are not available. TB enters the respiratory system and is phagocytosed by macrophages that present antigens to T cells. Sensitized T cells secrete interferon-gamma, which play a central role in the formulation and maintenance of granulomas, maintain TB in a dormant state for decades.^[4]^ Radiological findings of previous TB infection, nodules and scars on chest radiography, are included as risk factors for reactivation of LTBI.^[1]^ These findings could reflect the formation of granuloma developed by TB. Thus, intervention for LTBI should also be considered before or during CBZ treatment, especially in patients with radiological findings of previous infection, similar to the individual described in the present report.^[1,5]^ If the mediation of granuloma by T-cells were disturbed by immunosuppression, TB could be activated from inside the granuloma. Other immunosuppressive states, such as organ transplantation, uncontrolled diabetes mellitus, HIV positivity and anti-tumor necrosis-alpha therapy for rheumatoid arthritis, have been included as risk factors for the reactivation of LTBI.^[1,11]^

In Japan, the number of LTBI notification was reported to be 7255 cases in 2017, which remained virtually unchanged over the previous 5 years. Among the newly identified LTBI cases in 2017, patients 65 to 74 years of age accounted for the largest proportion (1199/7255 [16.5%]), followed by those 45 to 54 (1111/7255 [15.3%]), and 55 to 64 (1018/7255 [14.0%]) years of age.^[12]^ A previous report describing the age distribution of PC reported that the incidence of PC turned upward at 50 years of age, which persisted until reaching a peak in the eighth decade of life.^[13]^ Thus, these reports showed that the age distribution of LTBI and PC in elderly individuals overlapped. As such, caution should be exercised in PC patients undergoing chemotherapy with strong myelosuppressive drugs such as CBZ.

## 4 Conclusion

In conclusion, careful management of LTBI before initiation of CBZ is needed because of its severe myelosuppressive properties and the incidence of LTBI overlapping with that of PC in elderly individuals.

## Author contributions

**Supervision:** Kazuhiro Yoshimura, Hirotsugu Uemura.

**Visualization:** Eri Banno, Yasunori Mori.

**Writing – original draft:** Mamoru Hashimoto, Takafumi Minami.

**Writing – review & editing:** Mamoru Hamaguchi, Saizo Fujimoto, Tomoki Takahashi, Takashi Kikuchi, Shogo Adomi, Takayuki Ohzeki, Nobutaka Shimizu, Masahiro Nozawa, Kazuhiro Nose.
